# Paromomycin is a more effective selection agent than kanamycin in Arabidopsis harboring the neomycin phosphotransferase II transgene

**DOI:** 10.1371/journal.pone.0325322

**Published:** 2025-06-25

**Authors:** Colton P. Goodman, Victoria N. Padgett, Elijah R. Evans, Albrecht G. von Arnim

**Affiliations:** 1 Department of Biochemistry and Cellular & Molecular Biology, The University of Tennessee, Knoxville, United States of America; 2 Graduate School of Genome Science and Technology, The University of Tennessee, Knoxville, United States of America; Cornell University, UNITED STATES OF AMERICA

## Abstract

Neomycin phosphotransferase II (*nptII*) is a selectable marker gene that is commonly used in plant molecular genetics and crop improvement, helping researchers to identify and select transgenically modified plants. The NPTII enzyme binds to and phosphorylates the aminoglycoside family of antibiotics, which are known translation inhibitors. Once the aminoglycoside is phosphorylated it is unable to bind to the ribosome and can no longer disrupt translation. Currently, the most widely used selection agent for screening NPTII expressing seedlings is kanamycin. Because the *nptII* transgene is frequently silenced epigenetically, kanamycin can be too toxic to seedlings that weakly express NPTII, leading to false negatives and making it harder to accurately identify transgenic plants. In this study we investigate the related aminoglycoside, paromomycin, as an alternative, non-lethal, selection agent to kanamycin across a series of transgenic Arabidopsis lines with varying NPTII expression. We investigated phenotypes of transgenic seedlings during and after antibiotic exposure. Seedling pigmentation and size are useful phenotypes for selecting seedlings with low *nptII* expression. Additionally, monitoring the photosynthetic efficiency and flowering time can help reduce the risk of false-positive results when treating seedlings with paromomycin.

## Introduction

The neomycin phosphotransferase II (*nptII*) gene is widely used as a selectable marker for transgenes in Arabidopsis and other plants. Specifically, the *nptII* gene is part of T-DNAs and transposons that serve as insertional mutagens in the Feldmann [1], WISC [2], CSHL [3], FLAGdb [4], and SALK [5] collections among others, as well as in numerous T-DNA expression cassettes such as pBin19 [6]. The presence of the *nptII* gene is typically scored by its expression, which confers resistance to certain aminoglycoside antibiotics, such as kanamycin. Kanamycin inhibits prokaryotic (chloroplast) ribosomes, causing chlorosis and bleaching. When *nptII* is expressed well, which is typically the case in primary transformants, kanamycin is a more robust selection agent than other aminoglycosides such as gentamycin or hygromycin, which are detoxified by resistance genes other than *nptII*.

Many plant strains that harbor *nptII* suffer from partial epigenetic silencing of the *nptII* gene expression. Often strongly resistant stains will give rise to progeny with progressively weaker kanamycin resistance [7,8]. Because kanamycin is highly toxic to sensitive seedlings, partial silencing of the *nptII* gene results in poor survival. This makes it difficult or impossible to rely on kanamycin as a selection agent when following the transgene in segregating families and other genetic pedigrees, to confirm the presence of the transgene, or rule out contamination of seed batches with nontransgenic seeds. Silencing is typically attributed to RNA interference and RNA-dependent DNA methylation triggered by double-stranded RNA transcripts that themselves arise from complex, inverted-repeat T-DNA loci [7,9,10]. Researchers have been advised to not rely on the antibiotic resistance gene when following the T-DNA [8]. Overall, kanamycin has proven to be too harsh for transgenic lines where *nptII* has been silenced, making it a suboptimal selection agent.

The *nptII* gene also confers resistance to the aminoglycoside paromomycin. Aminoglycoside antibiotics are characterized by a common 2-dexoystreptamine ring, which in kanamycin contains two substitutions in the 4,6 positions and in paromomycin has substitutions in the 5,6 positions. Like kanamycin, paromomycin binds to rRNA helix 44 in the A-site of the small ribosomal subunit, which is the tRNA decoding center of the ribosome [11,12]. While kanamycin is mainly active against prokaryotic ribosomes, paromomycin inhibits translation by binding to the prokaryotic 30S subunit [13] as well as to the decoding center in the eukaryotic 40S subunit [12]. In eukaryotes, paromomycin stimulates the misreading of tRNAs and subsequent misincorporation of amino acids. This effect can be seen on cytosolic ribosomes in the wheat germ *in vitro* translation system [14] and in an *in vitro* translation assay in cultured human cells [15]. Kanamycin A does not have this activity. In wheat germ, paromomycin only weakly inhibits amino acid incorporation [14]. Paromomycin also stimulates stop codon readthrough in mammalian cells in vivo [16,17], which is stop codon-dependent and sequence context-dependent [18]. Thus, paromomycin has a different activity profile from kanamycin. With a notable exception in plant tissue culture [19], paromomycin is not widely used as a selection agent for *nptII*.

We tested whether paromomycin may be a more benign selection agent than kanamycin in Arabidopsis seedlings harboring *nptII* transgenes at various levels of partial gene silencing. Here we demonstrate that paromomycin at the optimized concentration distinguishes *nptII*-positive from *nptII*-negative seedlings. Seedlings with weak kanamycin resistance have adequate resistance to paromomycin, which is evident by a larger size and less stress-induced anthocyanin. These plants can survive well after they have been removed from the paromomycin selection medium. However, because paromomycin only stalls growth but does not kill *nptII*-negative plants, care is needed to prevent the escape of truly sensitive plants. Selecting for the *nptII* gene using paromomycin is generally more benign than using kanamycin and can be used for plants with fully active or substantially silenced *nptII* expression.

## Results and discussion

### Paromomycin selects for Arabidopsis seedlings with weakly expressed nptII transgenes

**Fig 1:**
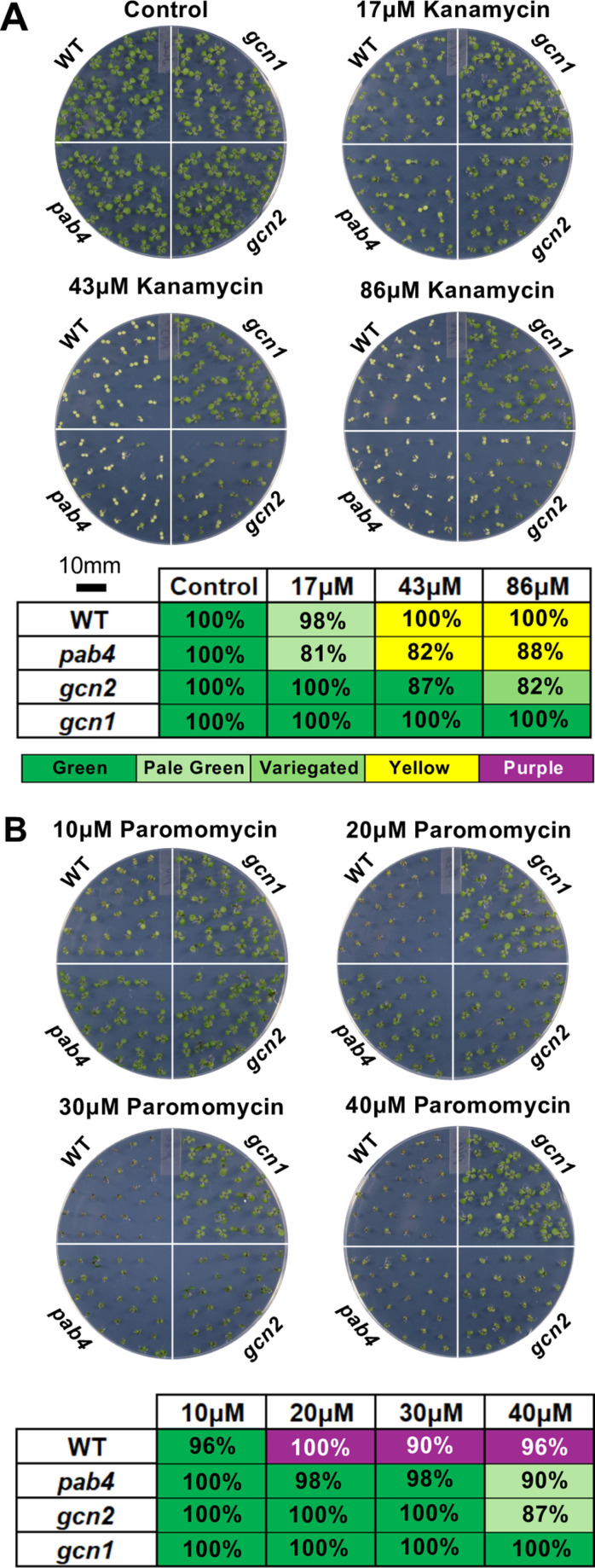
Paromomycin is a gentler selection agent than kanamycin for seedlings that weakly express the *nptII* transgene. Seedlings of three *nptII*-positive mutant lines and corresponding *nptII*-negative wild type (Columbia ecotype) were grown for seven days on ½ x MS medium with 1% sucrose containing the indicated concentrations of **(A)** kanamycin and **(B)** paromomycin. The graphic below each image summarizes the consensus phenotype under each condition with a rubric that focuses on seedling pigmentation. The percentages represent the proportion of seedlings within the sample population exhibiting the pigmentation phenotype indicated in the box. Each sample has about 25 seedlings, and the experiment was replicated at least three times.

**Fig 2:**
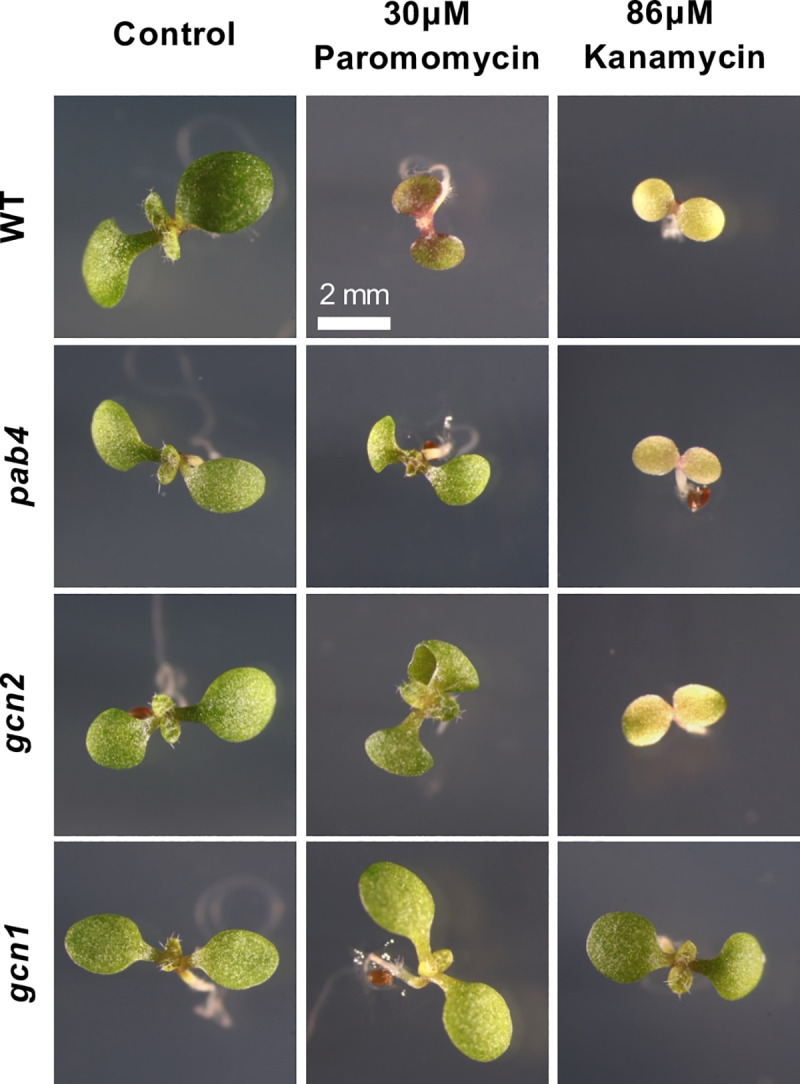
Paromomycin acts as a less harsh selection agent than kanamycin for weakly resistant seedlings. Representative close-up images of 7-day-old Arabidopsis seedlings grown on ½ strength MS medium with 1% sucrose. The chosen mutants are all from the SALK T-DNA collection and are compared to their wild-type (WT) Columbia. Optimal concentrations of kanamycin and paromomycin were added. All images are at the same magnification.

The neomycin phosphotransferase (*nptII)* confers resistance to kanamycin but Arabidopsis strains harboring a *nptII* transgene vary dramatically in the extent of their kanamycin resistance. We chose three *nptII*-transgenic insertion mutants, one allele each for the translational regulator genes *GENERAL CONTROL NONDEREPRESSIBLE1* and *2 (GCN1* and *GCN2)*, and an allele of POLY(A)-BINDING PROTEIN4 (PAB4). Under the typical concentration of 50 mg/L (86μM), resistance may be complete as in the case of the *gcn1* mutant, partial, as exemplified by green-yellow variegation on the cotyledons *in gcn2*, or essentially negligible as demonstrated in *pab4* (Fig 1A). The variable resistance observed in these strains was directly correlated to the expression of NPTII protein (S1 Fig). However, on 30μM paromomycin, all three mutants showed some degree of growth advantage compared to the corresponding wild type, ecotype Columbia (Fig 1B). Characteristic phenotypes of individual seedlings are shown in Fig 2. On paromomycin, the wild type remained green and did not bleach out, but expressed purple anthocyanin pigments abundantly. Weakly resistant seedlings such as *pab4* had larger cotyledons than wild type, less anthocyanin, and their primary leaves emerged sooner (Fig 2). These results show that paromomycin is a milder selection agent than kanamycin, which enables identification of seedlings with weak, largely silenced kanamycin resistance.

We explored the dose-response characteristics of both kanamycin and paromomycin. Using lower concentrations of kanamycin can be a solution (*gcn2* in Fig 1A). However, identifying the ideal dose of kanamycin is not practical, given that researchers who receive a new strain with unknown *nptII* expression often receive very few seeds. Lowering the dose of paromomycin allowed both wild type and *nptII*-positive lines to grow faster, while raising it to 40μM was not advantageous (Fig 1B). Over the course of many experiments, 30μM paromomycin resulted in an acceptable and reproducible difference between wild type and mutants; in combination, the larger size of the cotyledons, lesser hyponasty (‘curled-under’), greener and less purple pigmentation of the cotyledons, and the earlier emergence of primary leaves are four phenotypes that together help to identify weakly *nptII*-expressing seedlings on paromomycin.

### Related seedling phenotypes on paromomycin

Neither of the antibiotics affected the seedling’s ability to germinate at any concentration (S2 Fig). While our medium routinely contained 1% sucrose, paromomycin also selected well on plates lacking sucrose (S3 Fig). However, because seedlings grow more uniformly and vigorously when sucrose is included, adding sucrose makes it easier to distinguish wild type from marginally resistant seedlings.

**Fig 3:**
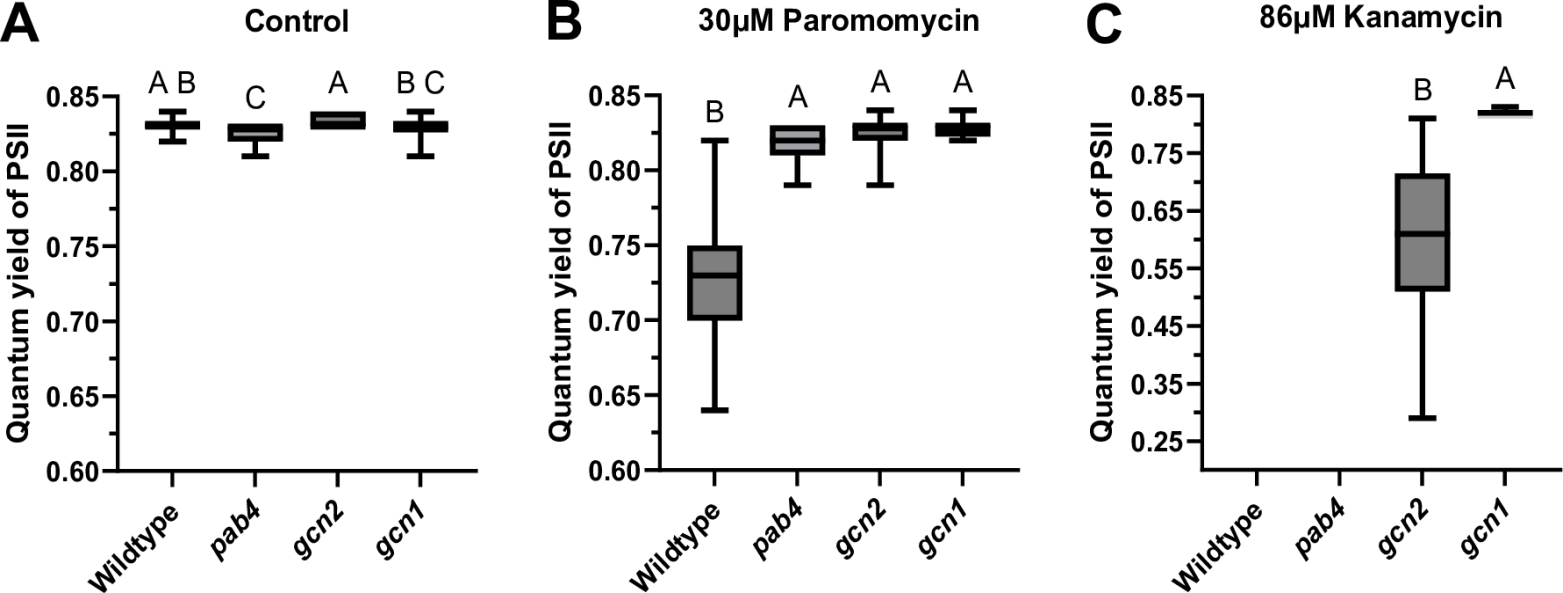
Paromomycin resistant seedlings maintain the quantum yield of photosystem II photosynthesis. The quantum yield of photosystem II is defined as the ratio of variable fluorescence over maximal fluorescence (Qy_max_ = F_v_/F_m_) when dark-adapted seedlings are exposed to a saturating pulse of light using the Fluorocam. Panels **(A)**, **(B)**, and **(C)** display the same four genotypes under the indicated antibiotic conditions. Each sample has about 25 seedlings. Error bars indicate standard deviations. An ordinary one-way ANOVA with a Tukey’s multiple comparison test was done to compare the genotypes. Data series that share the same letter are not significantly different.

The pigmentation observed in the cotyledons reflects the ability of seedlings to perform processes such as photosynthesis. On paromomycin the quantum yield of photosystem II (PSII) was decreased in wild-type seedlings but maintained in all NPTII transgenic strains (Fig 3A, B). Furthermore, the harshness of kanamycin was reflected by a decrease of the quantum yield in *gcn2* and no measurable data in *pab4* and wild type (Fig 3C).

**Fig 4:**
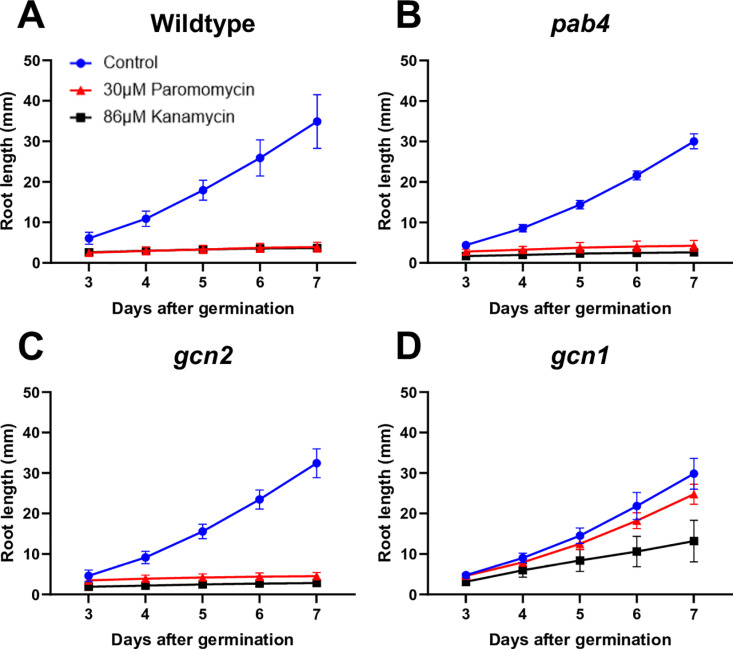
Root length is useful in selecting for strongly resistant seedlings only. Root lengths were measured daily for seedlings grown on vertical plates containing ½ strength MS media with 1% sucrose as the control (blue). Antibiotic treatments consisted of 30μM paromomycin (red) and 86μM kanamycin (black). **(A)** Wild type. **(B)** *pab4*. **(C)** *gcn2-2*. **(D)** *gcn1-5*. Error bars indicate standard error of the mean.

We tested whether root length scored on vertical plates would be informative in revealing paromomycin resistance. However, in moderately or weakly resistant strains such as *gcn2* and *pab4*, the roots were too sensitive to paromomycin to differentiate mutants from wild type (Fig 4A-D).

S4 Fig demonstrates that paromomycin is effective for selecting NPTII positive *gcn2* seedlings out of segregating families where kanamycin resistance is variable, weak or absent.

One might hypothesize that paromomycin resistance may be a phenotype of certain Arabidopsis mutations that affect some aspect of translation, given that paromomycin inhibits translation. GCN1, GCN2, and PAB4 are all implicated in translational control. However, NPTII positive mutants in genes that bear no relation to the translation machinery also express paromomycin resistance (S5 Fig), if they harbor *nptII*.

### Adult plant phenotypes on paromomycin

**Fig 5:**
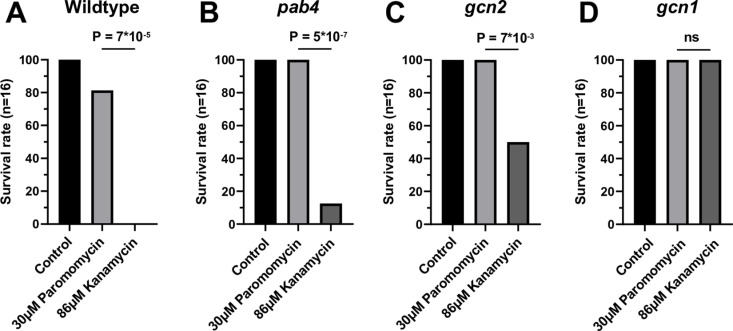
Survival of seedlings on soil depends on the prior antibiotic treatment. Seedlings of four genotypes were germinated on the indicated concentrations of antibiotics and transplanted to soil on day 7. No antibiotic was applied after transplantation. Survival was scored as the ability of the plant to complete all stages of its life cycle and produce seeds. **(A)** Wild type. **(B)** *pab4*. **(C)** *gcn2-2*. **(D)** *gcn1-5*. The difference between paromomycin and kanamycin survival rates was evaluated using a Fisher’s exact test. Data represent one experiment out of three biological replicates. Not significant (ns).

On paromomycin the seedling phenotypes do not distinguish resistant seedlings from wild type as clearly as on kanamycin. Therefore, we compared how adult plant phenotypes, rosette growth, flowering time, and overall survival were affected by the two antibiotics. Of note, *nptII* negative seedlings such as wild type that were germinated on 30μM paromomycin did recover and survive when transplanted to soil, whereas wild-type seedlings exposed to kanamycin generally did not survive (Fig 5A). This disadvantage of paromomycin was compensated because weakly resistant *pab4* and *gcn2−2* mutants survived after paromomycin but not kanamycin (Fig 5B-D).

**Fig 6:**
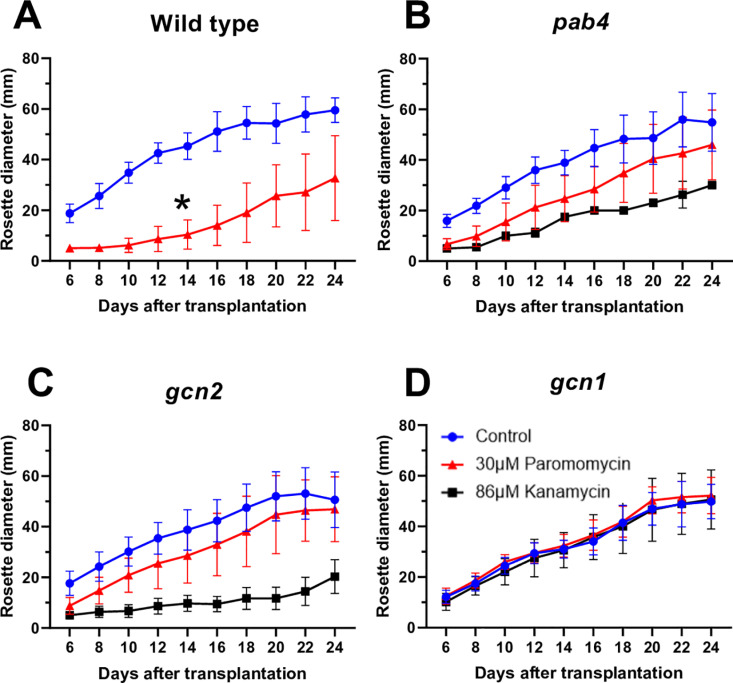
Vegetative rosettes grow faster in plants with weak paromomycin resistance than in false positives. Seedlings of four genotypes were germinated on the indicated concentrations of antibiotics and transplanted to soil on day 7. Rosette diameters were measured from n = 16 plants using a standard ruler starting on day 6 after transplantation until they were fully grown. Error bars indicate standard deviations. **(A)** Wild type. **(B)** *pab4*. **(C)** *gcn2-2*. **(D)** *gcn1-5*. Note that the growth rate of paromomycin-treated wild type (A) is strongly reduced compared to *pab4* (B) and *gcn2-2* (C) and *gcn1-5* **(D)**.

As paromomycin-treated seedlings are transplanted to soil, the growth of the vegetative rosette is another indicator of resistance. Seedlings with poorly expressed *nptII* and thus mild paromomycin resistance recovered quickly despite the antibiotic and grew with just a slight delay as compared to the control (no antibiotic) (Fig 6B,C). In contrast, wild-type seedlings recovered more slowly (Fig 6A), similar to seedlings selected on kanamycin (Fig 6B, C). Of course, fully resistant seedlings such as *gcn1–5* grew rapidly, without any delay (Fig 6D).

**Fig 7:**
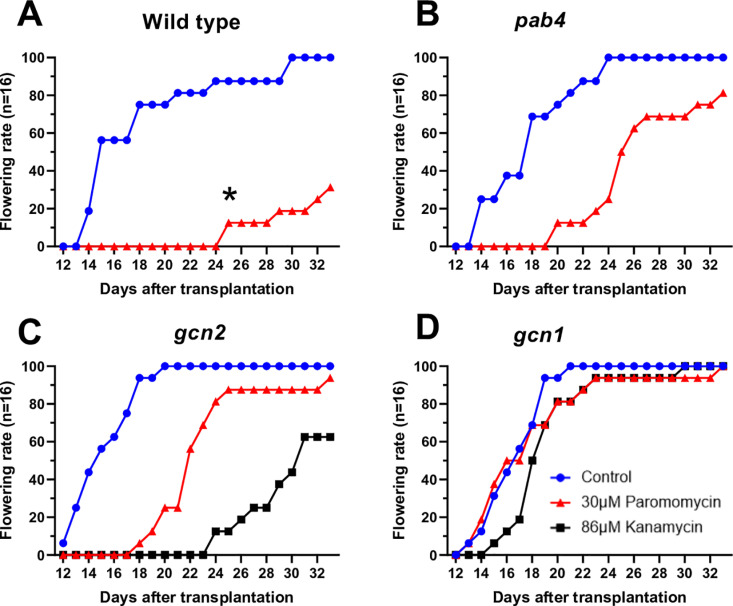
Plants with weak paromomycin resistance flower earlier than non-transgenic false-positives. Seedlings of four genotypes were germinated on the indicated concentrations of antibiotics and transplanted to soil on day 7. Plants were scored as flowering once the inflorescence began to emerge from the center of the rosette. Data are cumulative traces from n = 16 plants per line. **(A)** Wild type. **(B)** *pab4*. **(C)** *gcn2−2*. **(D)** *gcn1−5*. Note that the flowering of paromomycin-treated wild type (A) was delayed compared to *pab4* **(B)**, *gcn2−2* **(C)**, and *gcn1−5* **(D)**. Pretreatment of the moderately resistant *gcn2−2* with kanamycin also strongly delayed flowering; kanamycin-treated *pab4* did not reach the flowering stage in this experiment.

Finally, flowering time can be used as an indicator of paromomycin resistance. Weakly resistant plants such as *pab4* and *gcn2−2* did flower later than strongly resistant plants (Fig 7B,C), but not as late as surviving wild type, most of which had not flowered 30 days after transplanting (Fig. 7A). Again, strongly resistant plants did not delay their flowering (Fig. 7D).

## Conclusion

If the *nptII* transgene is weakly expressed in Arabidopsis thaliana, exposure of seedlings to kanamycin as a selection agent will harm the plant to the extent that selection will often be impossible. The use of kanamycin on strains that weakly express the NPTII enzyme can often lead to false-negative results. Here we showed how genotypes that weakly express *nptII* can be selected successfully on 30μM paromomycin. Paromomycin is a milder reagent. While this avoids the problem of false-negative results, it can cause false-positives, but this problem can be managed. Compared to kanamycin, where greening alone is sufficient to distinguish resistant seedlings, several phenotypes should be taken into account with paromomycin. Sensitive seedlings contain more anthocyanin than weakly resistant ones. In addition, sensitive seedlings are generally smaller, have more hyponastic cotyledons, and their primary leaves emerge more slowly. After transplanting candidates to soil, sensitive ‘escapes’ (false-positives) will grow more slowly and flower later than even weakly resistant seedlings. Altogether, using paromomycin as a selection agent does not risk the life of the seedling. It can greatly speed up the identification of *nptII*-containing transgenic plants as compared to the next best alternative, genotyping for the transgene using polymerase chain reaction.

## Materials and methods

### Arabidopsis strains

Arabidopsis thaliana strains included homozygous *gcn1−5* (*ila-3*, CS65594, [20]), *gcn2−1* mutants of the GT8359 gene trap line [21], and homozygous *gcn2−2* (SALK_032196) [22]; and *gcn2−3* (SALK_129334.2) [23] and *pab4* (SALK_113383) [24,25], the *xrn2−1* allele SALK_041148 in strain *xrn2−1, xrn3−3* [26], the *fry1−6* allele SALK-020882 of *SAL1/FRY1* [27], as well as corresponding ecotype Landsberg (Ler-0, for gcn2−1), Columbia (Col-0, other mutants).

### Growth conditions

Seeds were surface-sterilized in 30% household bleach with 0.1% Triton-X-100 for 10−15 minutes and then washed with sterile water four times. Seedlings were spotted on solid germination medium containing ½ strength MS salts (MP Biomedicals, cat # 2633024), 0.5g/L MES buffer pH 5.7, 1% sucrose and 10% Phytoagar (Bioworld, cat # 40100072−2). Kanamycin (Acros Organics Cat No. AC450810100) was dissolved in water at 50 mg/mL, and paromomycin (Sigma-Aldrich, Cat No. P9297-1G) was dissolved in water at 18 mg/mL. The stock of paromomycin was stable when stored at -20^o^C for at least 3 months. Once added to the medium and stored in the dark at 4^o^C, the paromomycin gave consistent results for at least 3 months. Seeds were stratified at 4^o^C for two days. Seedlings were moved to an incubator set to a constant 22^o^C on a 16-hour-light 80 μEin m^-2^ s^-1^/8-hour-dark cycle, marking the beginning of germination. Seedlings were transplanted to soil seven days after germination to score rosette diameter and flowering time. Germination was scored as the emergence of the radicle from the seed coat. Flowering was scored as the emergence of the inflorescence in the center of the vegetative rosette.

### Immunoblotting

The expression level of NPTII protein was characterized by immunoblotting of protein extracts separated by SDS-polyacrylamide gel electrophoresis from 10-day-old seedlings using as antibody the mouse anti-neomycin phosphotransferase II from NOVUS catalog # NB110–60487 at a concentration of 1:5000. Gels were blotted using a Biorad wet-transfer apparatus with transfer buffer (25mM Tris, 192mM glycine, 20% methanol). After transfer to PVDF, the membrane was blocked in 1x Tris-buffered saline (25mM Tris, 150mM NaCl, 2.5mM KCl) with 2% bovine serum albumin and 0.2% Tween 20. Blots were developed with 1:20000 anti-mouse IgG coupled to horseradish peroxidase (R&D systems, catalog # HAF007) as secondary antibody and the SuperSIgnal West Pico PLUS Chemiluminescent Substrate (catalog # 34577). Blots were imaged on the Biorad ChemiDoc MP imager and digital signals were quantified with Fiji Image J software.

### Photosynthetic efficiency measurement

The maximum quantum yield of photosystem II (Qymax = F_v_/F_m_) was measured on a FluorCam 800MF (Photon Systems Instruments, Drásov, Czechia) as per the manufacturer’s instructions and modifications [28]. Briefly, plants were dark adapted for 10 min (F_0_) prior to applying a saturating pulse of 1800 μEin m^–2^ s^–1^ for 0.8 s (F_m_). Variable fluorescence (F_v_) was calculated as the difference between baseline fluorescence F_0_ and maximal fluorescence F_m_ to obtain the maximum quantum yield (F_v_/F_m_).

### Accession numbers

The Arabidopsis locus identifiers for the wild type genes are as follows: *GCN1* is At1g64790, *GCN2* is At3g59410, *PAB4* is At2g23350, XRN2 is At5g42540, and SAL1/FIERY1 is At5g63980.

## Supporting information

S1 Fig NPTII expression is correlated to kanamycin resistance. **(A)** NPTII immunoblots of seedling proteins from three replicate sets of plants of the indicated genotypes. The stochastic variation in signal between replicates is typical for transgenes subject to silencing. The image labeled Loading shows a section of a Coomassie-stained replicate gel to test for equal protein loading in each lane. **(B)** Graph summarizing the NPTII expression from blots such as that in (A). Data are averages and standard deviation from n = 6–9 bands quantified using Fiji ImageJ. The strongest signal was set to 100% and the remainder were prorated accordingly and plotted on a log scale. Two times the standard deviation of six background intensities reflects the noise of the data and is reflected in the graph by the dotted line. A one-way ANOVA with a Tukey’s multiple comparison test was done to compare the genotypes. Data series that share the same letter are not significantly different.
(PDF)

S2 Fig Seedling germination is not affected by kanamycin or paromomycin. Germination percentages of **(A)** wild-type, **(B)** *pab4*, **(C)** *gcn2–2*, and **(D)** *gcn1–5* seeds treated with varying concentrations of kanamycin (dark grey) and paromomycin (light grey). The control conditions (black) consisted of ½ MS with 1% sucrose. Germination was recorded as emergence of the radicle from the seed coat four days after seeds had been moved into the light.
(PDF)

S3 Fig Selection with paromomycin in the absence of sucrose in the medium. Media consist of ½ x MS with 0.5g MES and 0% sucrose. Images were taken seven days after seed germination.
(PDF)

S4 Fig Selection under conditions of Mendelian segregation. We generated F2 families in which the *gcn2−2* allele segregated as a Mendelian trait. **(A)** Using paromomycin, the ratio of resistant to sensitive seedlings reached as high as the theoretically expected 3.0. In contrast, kanamycin resistance was too weak and variable to reliably identify resistant F2 seedlings. A Chi-square test was performed to determine if the expected 3:1 ratio of resistant to sensitive seedlings was observed. Such families are marked with *. **(B)** Representative view of a family on paromomycin segregating for resistant (large) and sensitive (small) seedlings.
(PDF)

S5 Fig Paromomycin remains effective when screening *nptII* transgenic mutants that do not affect translation. **(A)** Seedlings were grown on 1% sucrose media with the additives as listed. Seedlings are 10-days post germination. **(B)** 30μM Paromomycin **(C)** 86μM Kanamycin. The lines shown are a transgenic line harboring a yellow fluorescent protein fusion with Arabidopsis protein SALT TOLERANCE HOMOLOG (STH, At2g31380, [29]), a mutant of ribonuclease XRN2 (At5g42540), and a mutant of the 3’(2’),5’-bisphosphate nucleotidase SAL1/FIERY1 (At5g63980, [27]).
(PDF)

S6 Fig Original immunoblots for NPTII protein. Related to Supplementary Figure 1.
(PDF)

S7 File Raw numerical data used to generate the graphs in this article. (XLSX)
