# Supplementary material for: Paromomycin is a more effective selection agent than kanamycin in Arabidopsis harboring the neomycin phosphotransferase II transgene
Source: PLoS One. 2025 Jun 25;20(6):e0325322. doi: 10.1371/journal.pone.0325322 (PMC12193802; doi:10.1371/journal.pone.0325322)
Supplement: S1 Fig — (A) NPTII immunoblots of seedling proteins from three replicate sets of plants of the indicated genotypes. The stochastic variation in signal between replicates is typical for transgenes subject to silencing. The image labeled Loading shows a section of a Coomassie-stained replicate gel to test for equal protein loading in each lane. (B) Graph summarizing the NPTII expression from blots such as that in (A). Data are averages and standard deviation from n = 6–9 bands quantified using Fiji ImageJ. The strongest signal was set to 100% and the remainder were prorated accordingly and plotted on a log scale. Two times the standard deviation of six background intensities reflects the noise of the data and is reflected in the graph by the dotted line. A one-way ANOVA with a Tukey’s multiple comparison test was done to compare the genotypes. Data series that share the same letter are not significantly different. (PDF) [file pone.0325322.s001.pdf]

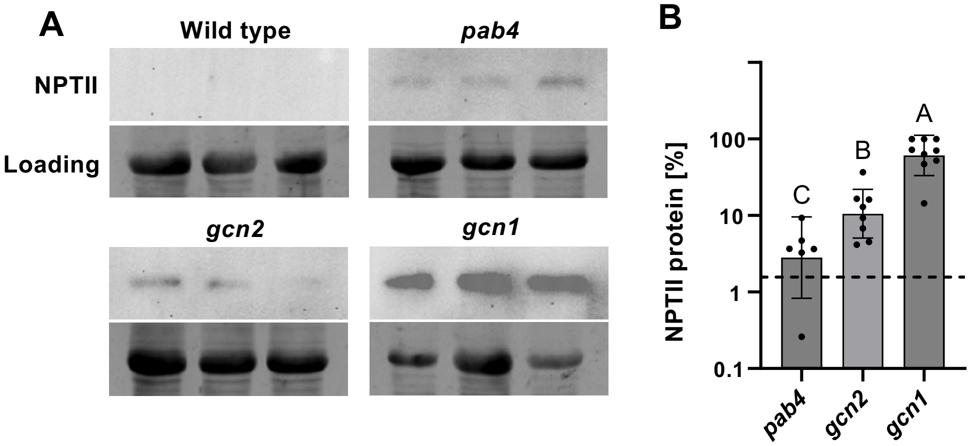


***Supplemental Figure 1: NPTII expression is correlated with kanamycin resistance. (A)*** NPTII *immunoblots of seedling proteins from three replicate sets of plants of the indicated genotypes. The stochastic variation in signal between replicates is typical for transgenes subject to silencing. The image labeled Loading shows a section of a Coomassie-stained replicate gel to test for equal protein loading in each lane.* ***(B)*** *Graph summarizing the* NPTII *expression from blots such as those in (A). Data are averages and standard deviation from n=6-9 bands quantified using Fiji ImageJ. The strongest signal was set to 100% and the remainder were prorated accordingly and plotted on a log scale. Two times the standard deviation of six background intensities reflects the noise of the data and is indicated in the graph by the dotted line. A one-way ANOVA with a Tukey's multiple comparison test was done to compare the genotypes. Data series that share the same letter are not significantly different.*
