# Supplementary material for: Paromomycin is a more effective selection agent than kanamycin in Arabidopsis harboring the neomycin phosphotransferase II transgene
Source: PLoS One. 2025 Jun 25;20(6):e0325322. doi: 10.1371/journal.pone.0325322 (PMC12193802; doi:10.1371/journal.pone.0325322)
Supplement: S2 Fig — Germination percentages of (A) wild-type, (B) pab4, (C) gcn2–2, and (D) gcn1–5 seeds treated with varying concentrations of kanamycin (dark grey) and paromomycin (light grey). The control conditions (black) consisted of ½ MS with 1% sucrose. Germination was recorded as emergence of the radicle from the seed coat four days after seeds had been moved into the light. (PDF) [file pone.0325322.s002.pdf]

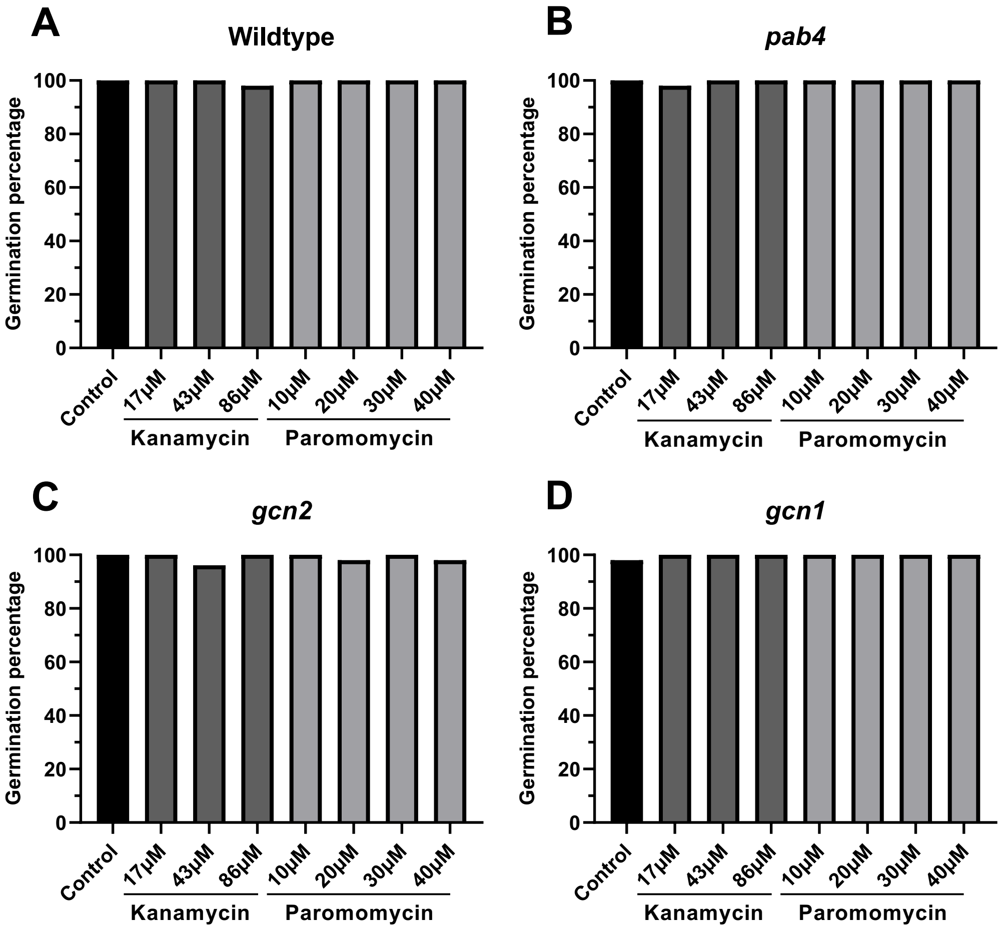

Supplemental Figure 2. Seedling germination is not affected by kanamycin or paromomycin. *Germination percentages of* ***(A)*** *wild-type,* ***(B)*** *pab4,* ***(C)*** *gcn2-2, and* ***(D)*** *gcn1-5 seeds treated with varying concentrations of kanamycin (dark grey) and paromomycin (light grey). The control conditions (black) consisted of ½ MS with 1% sucrose. Germination was recorded as emergence of the radicle from the seed coat four days after seeds had been moved into the light.*
