# Supplementary material for: Paromomycin is a more effective selection agent than kanamycin in Arabidopsis harboring the neomycin phosphotransferase II transgene
Source: PLoS One. 2025 Jun 25;20(6):e0325322. doi: 10.1371/journal.pone.0325322 (PMC12193802; doi:10.1371/journal.pone.0325322)
Supplement: S3 Fig — Media consist of ½ x MS with 0.5g MES and 0% sucrose. Images were taken seven days after seed germination. (PDF) [file pone.0325322.s003.pdf]

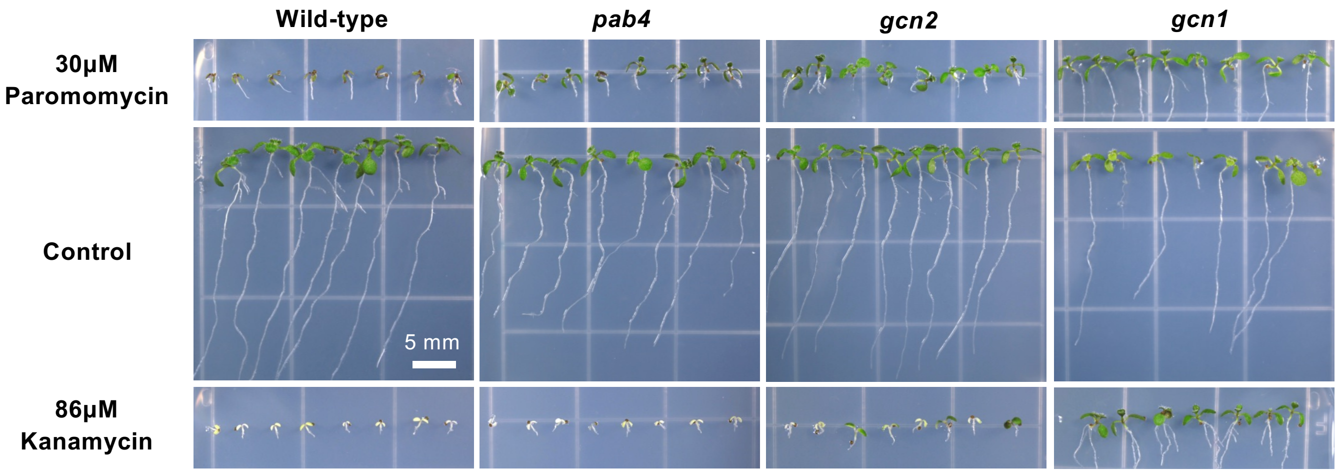

Supplemental Figure 3. Selection with paromomycin in the absence of sucrose in the medium. *Media consist of ½ x MS with 0% sucrose. Images were taken seven days after seed germination.*
