# Supplementary material for: Paromomycin is a more effective selection agent than kanamycin in Arabidopsis harboring the neomycin phosphotransferase II transgene
Source: PLoS One. 2025 Jun 25;20(6):e0325322. doi: 10.1371/journal.pone.0325322 (PMC12193802; doi:10.1371/journal.pone.0325322)
Supplement: S4 Fig — We generated F2 families in which the gcn2−2 allele segregated as a Mendelian trait. (A) Using paromomycin, the ratio of resistant to sensitive seedlings reached as high as the theoretically expected 3.0. In contrast, kanamycin resistance was too weak and variable to reliably identify resistant F2 seedlings. A Chi-square test was performed to determine if the expected 3:1 ratio of resistant to sensitive seedlings was observed. Such families are marked with *. (B) Representative view of a family on paromomycin segregating for resistant (large) and sensitive (small) seedlings. (PDF) [file pone.0325322.s004.pdf]

A

	Resistant	Sensitive	Ratio	
	**30μM Paromomycin**			
**WT**	0	60	NA	
gcn2-2	67	4	NA	
**Segregating**	84	26	3.23	*
**Segregating**	64	31	2.06	*
**Segregating**	61	18	3.39	*
	**86μM Kanamycin**			
**WT**	0	49	NA	
gcn2-2	0	68	NA	
**Segregating**	30	64	0.47	
**Segregating**	0	89	0	
**Segregating**	0	39	0	

B

30μM Paromomycin

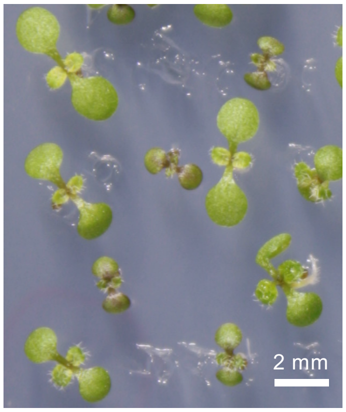

Supplemental Figure 4. Selection under conditions of Mendelian segregation. *We generated F2 families in which the gcn2-2 allele segregated as a Mendelian trait.* ***(A)*** *Using paromomycin, the ratio of resistant to sensitive seedlings reached as high as the theoretically expected 3.0. In contrast, kanamycin resistance was too weak and variable to reliably identify resistant F2 seedlings. A Chi-square test was performed to determine if the expected 3:1 ratio of resistant to sensitive seedlings was observed. Such families are marked with *.* ***(B)*** *Representative view of a family on paromomycin segregating for resistant (large) and sensitive (small) seedlings.*
