# Supplementary material for: Paromomycin is a more effective selection agent than kanamycin in Arabidopsis harboring the neomycin phosphotransferase II transgene
Source: PLoS One. 2025 Jun 25;20(6):e0325322. doi: 10.1371/journal.pone.0325322 (PMC12193802; doi:10.1371/journal.pone.0325322)
Supplement: S5 Fig — (A) Seedlings were grown on 1% sucrose media with the additives as listed. Seedlings are 10-days post germination. (B) 30μM Paromomycin (C) 86μM Kanamycin. The lines shown are a transgenic line harboring a yellow fluorescent protein fusion with Arabidopsis protein SALT TOLERANCE HOMOLOG (STH, At2g31380, [29]), a mutant of ribonuclease XRN2 (At5g42540), and a mutant of the 3’(2’),5’-bisphosphate nucleotidase SAL1/FIERY1 (At5g63980, [27]). (PDF) [file pone.0325322.s005.pdf]

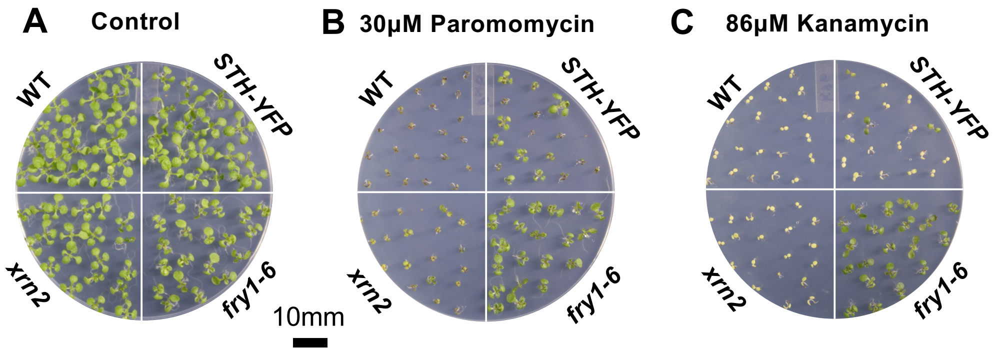

Supplemental Figure 5. Paromomycin remains effective when screening nptII transgenic mutants that do not affect translation. (A) *Seedlings were grown on 1% sucrose media with the additives as listed. Seedlings are 10-days post germination.* ***(B)*** *30μM Paromomycin* ***(C)*** *86μM Kanamycin. The lines shown are a transgenic line harboring a yellow fluorescent protein fusion with Arabidopsis protein SALT TOLERANCE HOMOLOG (STH, At2g31380, [30]), a mutant of ribonuclease XRN2 (At5g42540), and a mutant of the 3'(2'),5'-bisphosphate nucleotidase SAL1/FIERY1 (At5g63980, [27]).*
