# Supplementary material for: Paromomycin is a more effective selection agent than kanamycin in Arabidopsis harboring the neomycin phosphotransferase II transgene
Source: PLoS One. 2025 Jun 25;20(6):e0325322. doi: 10.1371/journal.pone.0325322 (PMC12193802; doi:10.1371/journal.pone.0325322)
Supplement: S6 Fig — Related to Supplementary Figure 1. (PDF) [file pone.0325322.s006.pdf]

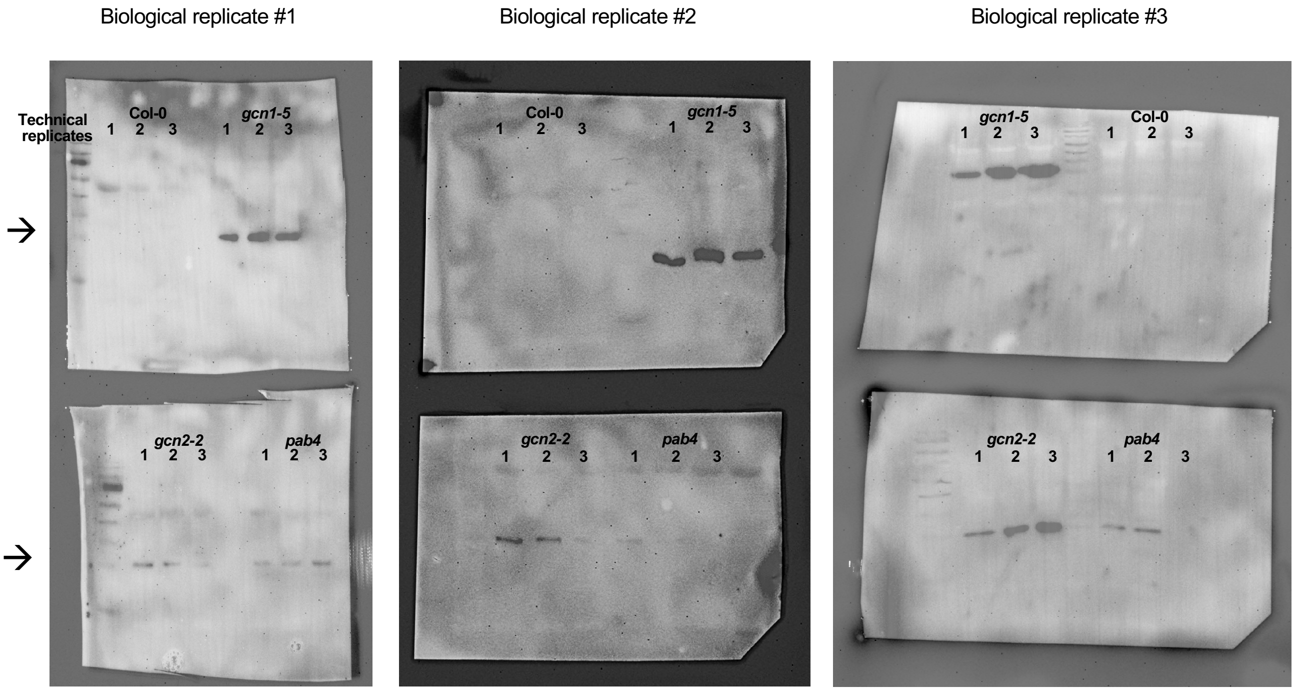

Supplemental Figure 6. Original immunoblots for NPTII protein. *Related to Supplementary Figure 1.*
